# Donor-derived *Cryptococcus gattii* complex infection after liver transplantation

**DOI:** 10.1590/0037-8682-0297-2024

**Published:** 2025-01-27

**Authors:** Bruno Hassunuma Carneiro, Giovanni Luis Breda, Regielly Caroline Raimundo Cognialli, Germana Davila dos Santos, Vania Aparecida Vicente, Eduardo Gnoatto Perondi, João Cesar Beenke França, Flávio Queiroz-Telles

**Affiliations:** 1 Universidade Federal do Paraná, Hospital de Clínicas, Curitiba, PR, Brasil.; 2 Universidade Federal do Paraná, Departamento de Clínica Médica, Programa de pós-graduação em Medicina Interna e Ciências da Saúde, Curitiba, PR, Brasil.; 3 Universidade Federal do Paraná, Departamento de Biotecnologia e Engenharia de Bioprocessos, Programa de pós-graduação em Biotecnologia e Engenharia de Bioprocessos, Curitiba, PR, Brasil.; 4 Universidade Federal do Paraná, Departamento de Patologia Básica, Programa de Microbiologia, Parasitologia e Patologia, Curitiba, PR, Brasil.; 5 University of Modena and Reggio Emilia, Modena, Itália.; 6 Universidade Federal do Paraná, Departamento de Saúde Coletiva, Curitiba, PR, Brasil.

**Keywords:** Cryptococcosis, C. gattii, Solid organ transplant

## Abstract

Cryptococcal disease is the third most common invasive fungal infection in solid organ transplant recipients and is associated with high-morbidity and -mortality rates. Donor-derived *Cryptococcus spp.* infection typically manifests within the first month post-procedure and has historically been caused by *C. neoformans* complex*,* predominantly in kidney recipients, but also after liver transplantation. We report the first documented case of donor-derived, early-onset *C. gattii* complex meningoencephalitis following liver transplantation in a 54-year-old woman, successfully treated with amphotericin B and fluconazole, and review the relevant literature.

## INTRODUCTION

Cryptococci are ubiquitous encapsulated yeasts that can cause severe invasive disease in humans and several other mammalian genera. Most cases are caused by two species complexes: *Cryptococcus neoformans*, typically affecting immunocompromised hosts, and *C. gattii*, which is more commonly associated with apparently immunocompetent patients^1^. Solid organ transplant recipients are at an increased risk of developing invasive fungal infections (IFIs), including candidiasis, aspergillosis, cryptococcosis, and other less common mycoses^2^. Among liver transplant recipients, the incidence of IFIs varies from 4-40%, and the mortality rate exceeds 25-60%^3^. Cryptococcal disease, mainly caused by *C. neoformans,* has been reported after liver transplantation, generally occurring several months to years post-procedure^4^ ^,^ ^5^. 

Herein, we report the case of a 54-year-old woman who developed early-onset *C. gattii* meningoencephalitis following liver transplantation. A comprehensive literature search was conducted in the Cochrane Library, LILACS, SciELO, Medline, PubMed, and PubMed Central databases. This case report is the first to document the development of donor-derived *C. gattii* complex infection following liver transplantation.

## CASE REPORT

**FIGURE 1:**
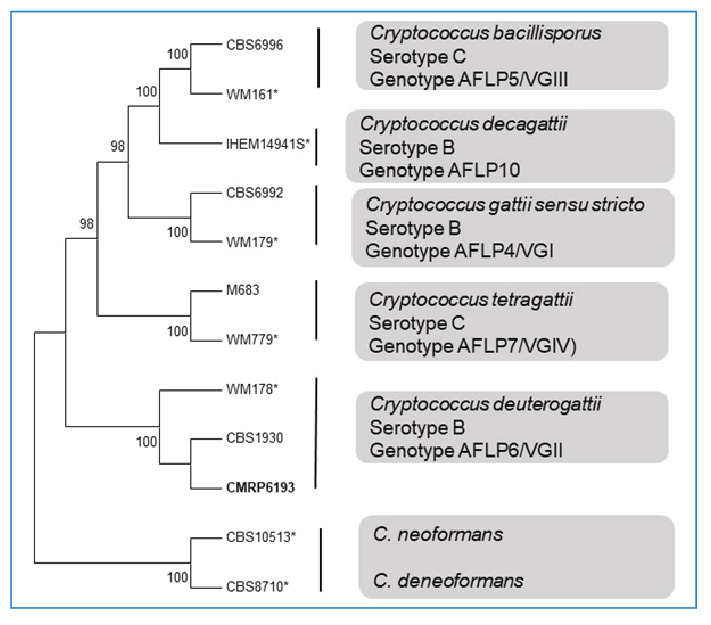
Maximum likelihood phylogenetic analysis with 1000 bootstrap replicates of *C. deuterogattii* (CMRP 6193) (in bold). The tree was performed based on the International Society for Human & Animal Mycology (ISHAM) consensus MLST. Bootstrap values are shown next to the branches, with only values >80 included. Reference strains (*).

A 54-year-old woman with a history of breast cancer, which was in remission, and porphyria cutanea tarda complicated by iron overload, cirrhosis, and hepatocellular carcinoma, underwent liver transplantation. The liver was donated by a kidney transplant recipient who died of suspected cytomegalovirus (CMV) encephalitis. During postoperative care, the patient received prophylactic ganciclovir for CMV and fluconazole (FLC) for *Candida* spp. She was discharged with an undetectable blood CMV viral load. One month later, the patient received high-dose glucocorticoid therapy for the treatment of acute rejection (confirmed by histological analysis of the graft) and was maintained on tacrolimus, mycophenolate mofetil, and prednisone (10 mg/day). Four days later, she was readmitted to the hospital due to fever, headache, and vomiting. Upon physical examination, the patient was conscious, alert, and had no neurological abnormalities. A head computed tomography (CT) scan was performed and revealed no abnormalities. Subsequently, a lumbar puncture was performed. The opening pressure was 30 cmH_2_O. Cerebrospinal fluid (CSF) analysis revealed neutrophilic pleocytosis (112 leukocytes/mm^3^, neutrophils 66%), elevated protein (320 mg/dL) and lactate (7.2 mmol/L) levels, and very low glucose (3 mg/dL) levels. Rare, encapsulated yeast cells consistent with *Cryptococcus* spp. were observed on nigrosin-stained direct microscopic examination of the CSF (quantification: 0.1 yeasts/µL) and the cryptococcal antigen lateral flow assay (CrAg LFA; Immuno-Mycologics; IMMY; Norman, OK) yielded a positive result. Induction therapy with amphotericin B (AmB) deoxycholate (50 mg/day) and high-dose FLC (800 mg/day) was initiated. *Cryptococcus* spp*.* were cultured from CSF after 6 days of incubation and identified as belonging to the *C. gattii* complex using matrix-assisted laser desorption/ionization time-of-flight mass spectrometry (MALDI-TOF MS) (Bruker Daltonics, Billerica, MA, USA). The isolate was then subjected to multilocus sequence typing (MLST). The nuclear loci CAP59, GPD1, IGS1, LAC1, PLB1, SOD1, and URA5 were amplified and sequenced as previously described^6^. The isolate was identified as *C. deuterogattii* and deposited in the Microbiological Collections of Paraná Network (CMRP-Taxonline) CMRP6193 (Figure 1). The newly generated sequence has been deposited in the National Center for Biotechnology Information (NCBI) GenBank nucleotide database (PQ467994 to PQ467999).

**FIGURE 2:**
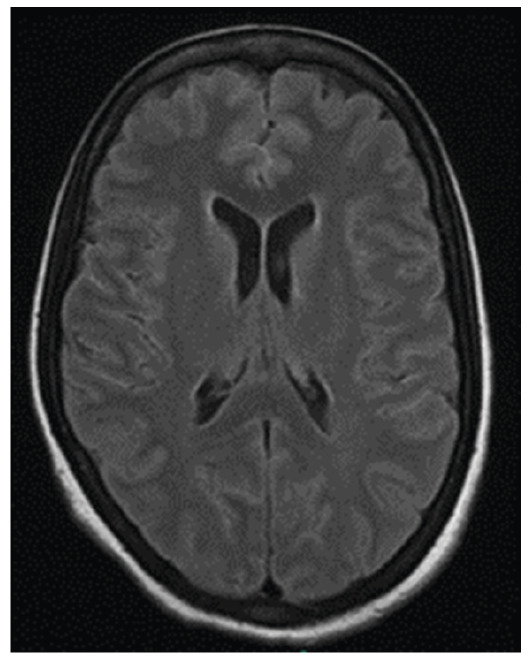
Head MRI revealing T2/FLAIR hyperintensities at the surface of the brain gyri and ependyma.

On the 11^th^ day of antifungal therapy, AmB was discontinued owing to the occurrence of renal toxicity, and high-dose FLC was continued as monotherapy. Follow-up CSF cultures revealed no fungal growth after the 13^th^ day of treatment. Multiple therapeutic lumbar punctures were performed to control the intracranial pressure. Magnetic resonance imaging (MRI) of the head revealed T2/FLAIR hyperintensities at the surface of the brain, cerebellar gyri, brainstem, ependyma and white matter, as well as leptomeningeal contrast enhancement (Figure 2). Chest CT performed to rule out pulmonary involvement revealed no signs of lung disease. The patient completed 8 weeks of antifungal therapy during hospitalization and received a 3-week course of intravenous ganciclovir due to the detectable CMV DNA in the CSF. Shortly after a diagnosis of cryptococcosis was made, a review of the donor’s cultures conducted at the originating institution revealed that *Cryptococcus* spp*.* had been identified in blood cultures, a result that was unavailable at transplantation. Due to persistent immunosuppression associated with treatment using everolimus, mycophenolate mofetil, and prednisone (5 mg/day), maintenance therapy with FLC (300 mg/day) was prescribed indefinitely. After 5 years of follow-up, the patient experienced no relapse of cryptococcal or CMV disease and showed no considerable sequelae.

## ETHICAL CONSIDERATIONS

The patient’s written consent for publication was obtained prior to data collection and making of this paper. This research has been approved by Hospital de Clínicas Federal University of Paraná Ethics Committee (research number 71396723.9.0000.0096).

## DISCUSSION

The classic pathophysiological mechanism of acquisition of cryptococcosis involves the inhalation of yeast cells and basidiospores, followed by late reactivation of latent infection. Under immunosuppressive conditions, such as HIV/AIDS or solid organ transplantation, the fungi can invade the bloodstream and disseminate to other organs, most commonly the central nervous system (CNS). This mechanism has been extensively studied in patients infected with *C. neoformans* ^1^. By contrast, *C. gattii* infections occur more frequently in apparently immunocompetent hosts and some studies suggest a particular preference for the lungs as the most common site of infection. The *C. gattii* complex is generally found in soil and trees, and comprises five species with different genotypes: *C. gattii sensu stricto* (VGI), *C. deuterogattii* (VGII), *C. bacillisporus* (VGIII), *C. tetragattii* (VGIV), and *C. decagattii* (VGVI)^1^ ^,^ ^7^. However, data on the full comprehension of *C. gattii* pathophysiological mechanisms, epidemiology, clinical characteristics, and optimal management remain scarce and are commonly derived from small observational studies and case reports on outbreaks and endemic regions^8^.

Cryptococcosis following solid organ transplantation is more common in kidney recipients than in liver recipients. It rarely occurs within the first few days post-transplant, which generally represents a donor-acquired infection. Notably, the disease more commonly develops several months to years after transplantation, often due to the reactivation of latent infection triggered by immunosuppression^2^ ^,^ ^5^. All cases of cryptococcosis in liver recipients reported in literature that were identified microbiologically to the species level have been caused by *C. neoformans*, except for one patient with fungemia, in whom *C. albidus* was isolated. The clinical manifestations can vary widely, depending on the site of infection. The most commonly reported presentations have been meningoencephalitis and lung disease. Other manifestations that have been described include abscesses, fungemia, osteomyelitis, empyema, cutaneous/subcutaneous disease, hepatic involvement, lymphadenopathy, peritonitis, electrolyte disorders, and hyperammonemia^4^ ^,^ ^5^ ^,^ ^9^.

Historically, a definitive diagnosis of cryptococcosis has been established through fungal culture. Other available diagnostic methods include direct microscopic examination and molecular tests, such as polymerase chain reaction (PCR), which are not widely available in low-income regions^1^. The CrAg LFA can be extremely helpful in the diagnosis; it has shown excellent sensitivity and specificity in the CSF (>96-99% and >97-99%, respectively) and serum (>96% and >97%, respectively). However, not all commercial assays can detect *C. gattii* infections^1^ ^,^ ^10^_._

Currently, no randomized controlled trials have specifically addressed the treatment of cryptococcosis in solid organ transplant recipients or *C. gattii* complex infections in any setting. Treatment guidance is based on extrapolated data from *C. neoformans* infections in patients with HIV and findings of observational studies. Guidelines suggest a 2-week AmB-based induction course, preferably liposomal AmB, combined with either 5-flucytosine (5-FC) or FLC (800-1200 mg/day). High-dose FLC monotherapy can serve as an alternative option. Following induction, an 8-week consolidation course with FLC (400-800 mg/day) is administered. Moreover, maintenance therapy with FLC (200 mg/day) is recommended for 6-12 months, or indefinitely, as secondary prophylaxis, depending on the degree of immunosuppression. The suggested antifungal treatment for *C. gattii* is identical to *C. neoformans*, but longer induction periods may be considered (4-6 weeks), especially if cryptococcomas are present^10^ ^,^ ^11^. The efficacy of single-dose liposomal AmB regimens has not been evaluated in solid organ transplant patients or patients with *C. gattii* infections^12^. Our patient received AmB-deoxycholate plus FLC due to the unavailability of liposomal AmB and 5-FC, and then monotherapy with FLC due to AmB-related toxicity. Therapeutic lumbar punctures for intracranial pressure management are also recommended as an essential part of treatment and were performed accordingly in this case^10^.

Currently, there are no recommendations for screening of solid organ transplant recipients or donors with CrAg, nor for antifungal prophylaxis against *Cryptococcus* spp. infection in these populations. There are no clinical trials evaluating this specific scenario^11^. In a retrospective study, the screening of liver recipients with serum CrAg had limited value: 12885 latex agglutination tests were required to identify nine positive patients, of whom five were asymptomatic and three, despite not receiving treatment, had favorable outcomes^13^. However, high-risk patients may benefit from FLC prophylaxis following liver transplantation to prevent *Candida* spp. infections. This strategy could indirectly benefit patients with latent or donor-derived cryptococcal infection, possibly reducing the likelihood of disease development or postponing the disease to develop after cessation of antifungal therapy, as observed in the case we presented. In this case, the donor’s diagnosis of invasive cryptococcosis was made retrospectively, only after the disease had already developed in the recipient, leaving no opportunity for extended prophylaxis.

Although glucocorticoids and mycophenolate have been previously associated with the development of cryptococcosis, the role of calcineurin inhibitors in the pathogenesis of disseminated disease is somewhat controversial, due to their synergistic antifungal activity against *Cryptococcus* spp. *in vitro* and their association with a lower incidence of CNS involvement and mortality^11^. 

## CONCLUSION

This case report highlights the importance of cryptococcal infection in liver transplantation and challenges in recognizing this infection in both recipients and donors. Timely diagnosis, prevention, and appropriate treatment of IFIs are paramount for reducing morbidity and mortality.
